# Design of facile technology for the efficient removal of hydroxypropyl guar gum from fracturing fluid

**DOI:** 10.1371/journal.pone.0247948

**Published:** 2021-03-04

**Authors:** Shiliang Xu, Mengke Cui, Renjie Chen, Qiaoqing Qiu, Jiacai Xie, Yuxin Fan, Xiaohu Dai, Bin Dong

**Affiliations:** 1 State Key Laboratory of Pollution Control and Resource Reuse, College of Environmental Science and Engineering, Tongji University, Shanghai, PR China; 2 Shanghai Investigation, Design & Research Institute Co. Ltd, Shanghai, PR China; 3 CNPC Research Institute of Safety & Environmental Technology Co. Ltd, State Key Laboratory of Petroleum Pollution Control, Beijing, PR China; 4 PetroChina Xinjiang Oilfield Company, Karamay, PR China; Nanjing Tech University, CHINA

## Abstract

With the increasing demand for energy, fracturing technology is widely used in oilfield operations over the last decades. Typically, fracturing fluids contain various additives such as cross linkers, thickeners and proppants, and so forth, which makes it possess the properties of considerably complicated components and difficult processing procedure. There are still some difficult points needing to be explored and resolved in the hydroxypropyl guar gum (HPG) removal process, e.g., high viscosity and removal of macromolecular organic compounds. Our works provided a facile and economical HPG removal technology for fracturing fluids by designing a series of processes including gel-breaking, coagulation and precipitation according to the diffusion double layer theory. After this treatment process, the fracturing fluid can meet the requirements of reinjection, and the whole process was environment friendly without secondary pollution characteristics. In this work, the fracturing fluid were characterized by scanning electron microscopy (SEM), Energy dispersive X-ray (EDX), X-ray diffraction (XRD) and Fourier transformed infrared (FTIR) spectroscopy technologies, etc. Further, the micro-stabilization and destabilization mechanisms of HPG in fracturing fluid were carefully investigated. This study maybe opens up new perspective for HPG removal technologies, exhibiting a low cost and strong applicability in both fundamental research and practical applications.

## 1. Introduction

With the increasing demands of modern industries, energy issues has always been a great concern to humankind [1–4]. What is worthy of more attention is that crude oil as an essential non-renewable resource occupies a significant meaning in the world’s energy supplies [5, 6]. To realize the enhancement of crude oil productivity, the fracturing technology has been widely used in the process of crude oil extraction [7, 8].

From the first use of fracturing fluid to boost crude oil production in 1947 to the present, it has undergone a huge evolution [9]. Of late, fracturing technology was used in more than 90% of crude oil recovery operations, which had significant economic benefits in the exploration of the remaining oil potential and the exploitation of tight oil [10]. As is well-known, the greatest shortcoming of fracturing fluids is that they will cause serious formation damage, which is an increasing serious issue with detrimental effects on production enhancement [11]. Moreover, some hydrophilic organic additives are difficult to remove from wastewater because of the variety of additives in the fracturing fluid and their complicated structures, such as biocides, thickeners, corrosion inhibitors, viscosity modifiers and other chemicals [12–14]. Unfortunately, fracturing liquid could also potentially be harmful to the groundwater and human health, which has become one of the current oilfield water pollution sources, as well as secondary pollution [15–17]. More explicitly, the aforementioned issues are the bottlenecks that must be faced for crude oil production.

The total amount of fracturing flowback fluid generated by the operations has increased sharply with the large-scale development of oilfields and the frequent industrial fracturing construction operations [18, 19]. More seriously, the fracturing technology always requires copious amounts of water resources, which is still a potential waste for precious resource [20, 21]. Presently, oxidation and flocculation processes are mostly adopted to the treatment of fracturing fluid throughout the world. For example, Linden et al. demonstrated the potential of an activated sludge mixed liquor to degrade guar under typical flowback conditions [22]. Lester et al. proposed a tailored treatment approach of the aeration/precipitation (and/or filtration) combining with biological treatment (to remove dissolved organic matter) followed by reverse osmosis desalination for flowback recycling in future fracturing operations [23]. Cath et al. introduced a novel application of forward osmosis (FO) facilitating water reuse for fracturing operation and reducing the need for an additional water source [24]. Yet, these examples revealed that complicated operation, high cost and secondary pollution are difficult points that could be faced for their practical treatment of fracturing fluid. In addition to these, during the treatment process of fracturing fluid, the residual guar gum also faced the problem of filters blocking, which leaded to the failure of the treatment process due to the poor removal effect of guar gum. Up to now, few works related to the in-depth study of guar gum properties in high-salt complex water environment have been reported. How to deal with the fracturing fluid has been a very important and urgent issue, and experts in different fields are committed to solving above problems.

Guar gum, a kind of environmentally friendly natural occurring polymer, is used as thickeners in fracturing fluid [25–27]. Further, physical, chemical and biological properties of guar gum are determined by its chemical structure present on its backbone [28–30]. Generally, HPG is modified on the basis of guar gum by nucleophilic substitution reaction to introduce polar hydroxypropyl with hydrophilicity, which is usually added to the fracturing fluids and widely used in practical engineering [31]. These fracturing fluids were normally prepared by mixing in appropriate amounts of surfactants such as anionic, cationic and non-ionic etc. Among them, polymers, including polyacrylamide (PAM) or polyacrylate polymers, not only increased the viscosity of wastewater but also enhanced the difficulty of analyzing related characteristics in high-salt complex water environment. In this sense, research on the micro-stabilization mechanism and destabilization technology of HPG is crucial to the treatment of fracturing fluid. Considering the efforts undertaken so far, there is still an urgent need to come up with a low-cost, facile and efficient technology for its large-scale treatment of fracturing fluid.

Herein, we designed a facile and efficient technology of "gel-breaking, coagulation, precipitation" for effective treatment of fracturing fluids. This novel approach without any further modification and tedious steps, avoiding the complex process involving in the advanced oxidation process or microbiological method and so on. During the whole experiment, potassium persulfate (KPS), polyaluminium chloride (PAC) and polyacrylamide (PAM) were used as breaker, inorganic flocculant and organic flocculant, respectively. These reagents with obvious advantages of lower-cost and easily available were much more conducive to actual operation of oilfield. In addition, chemical composition and bonding structures of simulated fracturing fluid-0 and simulated fracturing fluid were analyzed and studied in more detail. Besides, we deeply explored and studied the destabilization mechanism of HPG on the basis of DLVO (Derjaguin–Landau–Verwey–Overbeek) theory in order to explain the aggregation and dispersion behavior of colloidal particles in fracturing fluid. Finally, through coagulation and precipitation process of simulated fracturing fluid, it remarkably indicated that HPG achieved the great removal effect by analyzing the data of flocculation efficiency and TOC removal efficiency. We believe that the facile and economical method has excellent potential in practical oilfield application and provided it candidate for comprehensive applicability.

## 2. Experimental section

### 2.1. Materials

The original fracturing fluid was collected from the Klamayi oilfield of Xinjiang, China. Hydroxypropyl guar gum (HPG) was purchased from Shanghai Yuanye Biological Technology Co., Ltd. PAM (cationic type) was supplied from Xinxiang Jinghua Water Purification Material Co., Ltd. Chitosan was provided by Shanghai Qiangshun Chemical Reagent Co., Ltd. Polyaluminium chloride (PAC), sodium sulfide (Na_2_S) and sodium tetraborate decahydrate were obtained from Aladdin Chemical Co., Ltd, China. Sodium hydroxide (NaOH), potassium bicarbonate (KHCO_3_), magnesium chloride (MgCl_2_), potassium persulfate (KPS) and potassium chloride (KCl) were purchased from Sinopharm Chemical Reagent Co., Ltd. The other reagents were all of analytical reagent grade.

### 2.2. Preparation and gel-breaking process of simulated fracturing fluid

The base fluid was prepared by mixing different reagents (0.5 wt% KCl, 0.4 wt% HPG, 0.015 wt% K_2_S_2_O_8_ and 0.005 wt% NaOH) and ionic mother liquor. 4 wt% borax was used as crosslinker and the crosslinking ratio of base fluid and crosslinker of 10: 1. Of these, the ionic mother liquor was the simulated ionic environment (Table 1). The specific preparation process and gel-breaking process were as follows. First, 0.5 wt% KCl was added to the ionic mother liquor. After that, HPG (0.4 wt%) was added slowly to the mixture under agitation. Then, 0.015 wt% K_2_S_2_O_8_ (150 mg·L^−1^) and 0.005 wt% NaOH were added to the above-mentioned solution and then set aside for 15 min. Subsequently, the resulting solution and crosslinker were mixed homogeneously in a ratio of 10:1 by stirring magnetically. The process was carried out under the 90°C water bath condition for about 2 h (S1 Fig). After process was finished, the mixture was naturally cooled to room temperature. At this stage, pH of the mixture was approximately 6.5. Finally, the resulting supernatant liquid were named as simulated fracturing fluid (Table 2); that is, simulated fracturing fluid completed the gel-breaking process. Accordingly, the resulting supernatant liquid were named simulated fracturing fluid-0 if the breaker (K_2_S_2_O_8_) was not added to the mixed solution.

**Table 1:** Formulation of ionic mother liquor.

Reagents	Concentration (mg·L^−1^)
FeCl_3_**·**6H_2_O	340.8
Na_2_S	60.8
Na_2_SO_4_	198.8
Na_3_PO_4_	736
MgCl_2_	74.1
CaCl_2_	2497.5
KHCO_3_	271.7

**Table 2:** Properties of simulated fracturing fluid.

Parameters	Values
pH	6.50 ± 0.20
Viscosity (mPa·s)	2.09 ± 0.15
Turbidity (NTU)	81.00 ± 29.30
Median particle size (nm)	3500 ± 500

### 2.3. Coagulation and precipitation process of simulated fracturing fluid

Firstly, the as-prepared simulated fracturing fluid completed the gel-breaking process through the specific steps. Subsequently, inorganic flocculant (PAC, 600 mg·L^−1^) and organic flocculant (PAM, 50 mg·L^−1^) were added into the same beaker containing simulated fracturing fluid in turn. Besides, inorganic flocculant (PAC, 800 mg·L^−1^), organic flocculant (PAM, 50 mg·L^−1^) and natural polymer flocculation (chitosan, 500 mg·L^−1^) were added respectively to simulated fracturing fluid and mixed according to a set of requirements as the control groups. Then, the obtained mixture was rapidly stirred at 300 rpm for 1 min and subsequently mix evenly at 100 rpm for 10 min. After stirring, the above mixture was allowed to precipitate around 15 min.

### 2.4. Characterization

#### 2.4.1. Characterization of original fracturing fluid

The original fracturing fluid was placed in the refrigerator at −80°C for more than 24 h until the sample was frozen hard. Then, the sample was freeze-dried in a high vacuum on the TENLIN FD-1B-80 freeze-drying machine until the moisture was completely removed. Finally, the sample was placed in a dryer to get fairly good preservation. The surface microstructures of the sample were observed by field emission scanning electron microscopy (FE-SEM, FEI Nova Nano SEM 450). The Bruker EDS QUA·NTAX equipped with an Energy dispersive X-ray (EDX) detector was used to characterize the chemical composition. The change of sample surface properties was investigated using Fourier transform infrared spectroscopy (FTIR, Nicolet 5700) with the wavenumber ranging from 1000 to 4000 cm^−1^.

#### 2.4.2. Other characterization

X-ray photoelectron spectroscopy (XPS, Thermo VG ESCA LAB 250) was employed to monitor the surface chemical composition of simulated fracturing fluid-0 and simulated fracturing fluid. The binding energy at 284.8 eV of C 1s peak was used to calibrate binding energy of all the spectra. The viscosity was tested by a rotational rheometer (Mars 60). The molecular weight was performed by High performance liquid chromatography (HPLC, Agilent LC 1100). The pH and alkalinity were measured with a FE-20 pH detector and FE28-standard apparatus (Mettlertoledo, Switzerland), respectively. The turbidity was obtained by using a portable turbidimeter (HACH-2100Q). Median particle size was determined using a Zetasizer Nano S90 (Malvern Instruments Ltd, UK). Total organic content (TOC) was determined by TOC analyzer (Shimadzu, TOC-4200).

Average values of related research data are obtained through five parallel experiments to ensure the reliability of data such as flocculation efficiency, TOC content and so on.

The error range of relevant data were also presented in the form of error bars.

## 3. Results and discussion

### 3.1. Morphology and chemical composition

**Fig 1:**
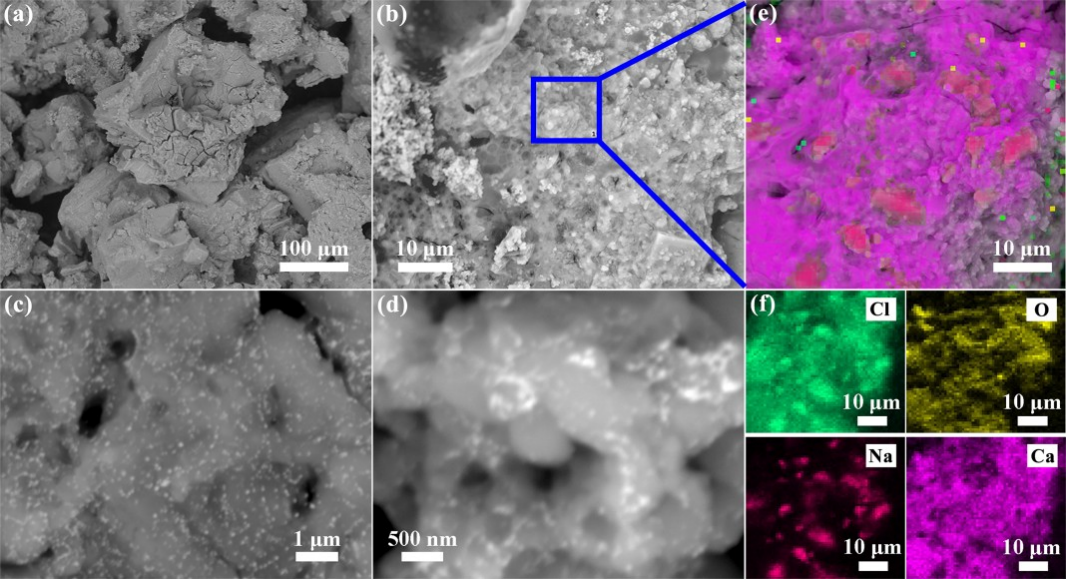
FE-SEM images of the original fracturing fluid at (a, b) low and (c, d) high magnifications. EDX Mapping results of the original fracturing fluid: (e) Mapping of all elements, (f) Mapping of Cl, O, Na and Ca.

The surface morphologies of the original fracturing fluid were characterized by FE-SEM. SEM images of original fracturing fluid, at different magnifications are shown in Fig 1A–1D. The overall structure was relatively loose and the particles were unevenly distributed with the magnification of 500 X, as exhibited in Fig 1A. As presented in Fig 1B of 4000 X magnification, the surface of the sample particles was not porous, presenting a unique structure similar to stalactite. In addition, and some crystal particles can also be seen on the surface of original fracturing fluid. At higher magnification of 30000 X and 60000 X, it was obviously observed that the guar gum exhibited a sticky state in Fig 1C and 1D. EDX analysis displayed a presence of high amounts of Cl, O, Na and Ca within the surface of original fracturing fluid (Fig 1E), and EDX mapping confirms the homogeneous nature of the specimen by showing the distribution of each element (Figs 1F and S2).

**Fig 2:**
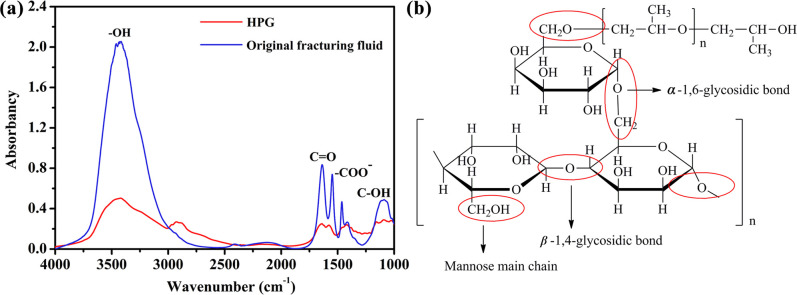
(a) FTIR spectra of the original fracturing fluid and pure HPG. (b) Schematic of broken keys for HPG.

The FT-IR spectrums of the original fracturing fluid and HPG are exhibited Fig 2A. The HPG exhibited characteristic broad absorption bands at 3423 cm^−1^ for O–H stretching, and the presence of absorption band at 2925 cm^−1^ was associated with C–H stretching vibrations. Additionally, the peaks at 1090 cm^−1^ were assigned to C–OH bending vibrations [32, 33]. In case of original fracturing fluid, the broad band around 3400 cm^−1^ was attributed to O–H stretching vibration [34, 35]. The absorption peaks at 1680 cm^−1^ and 1550 cm^−1^ were assigned to the stretching vibration of C = O and–COO–, respectively. We can also observe the C–OH stretching in the spectral regions between 1150 and 1000 cm^−1^. It can be inferred that the structure of HPG had been destroyed in the original fracturing fluid. Further, a significant increase in hydroxyl may be due to breaking of the bond of carbon-oxygen bond at position 1 of galactose unit, *α*-1, 6 glycosidic bond and *β*-1, 4 glycosidic bond, and some hydroxyl groups were also oxidized to carbonyl groups, depicted in Fig 2B [36]. The thickener in the original fracturing fluid was partially decomposed due to the influence of formation temperature and the presence of breaker. In other words, the HPG no longer existed in the form of cross-linked macromolecules. Besides, the complex component of original fracturing fluid might cause some interference to the analysis of the material structures.

### 3.2. Analysis of gel-breaking process

**Fig 3:**
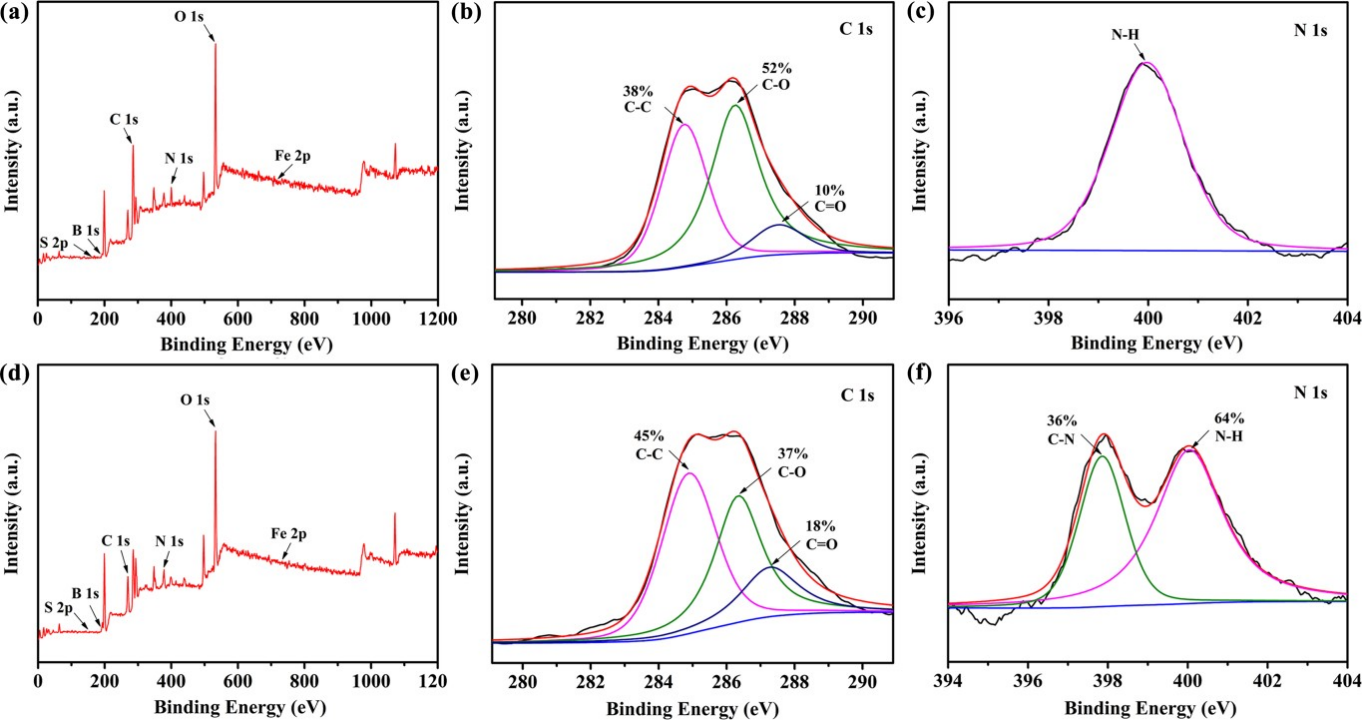
XPS full-scan spectrum of (a) simulated fracturing fluid-0 and (d) simulated fracturing fluid (after gel-breaking). High-solution C 1s XPS spectra of (b) simulated fracturing fluid-0 and (e) simulated fracturing fluid. High-solution N 1s XPS spectra of (c) simulated fracturing fluid-0 and (f) simulated fracturing fluid.

The composition of the original fracturing fluid was extremely complicated, and the structure of HPG will change when it was kept for a long period of time, affecting the properties of the original fracturing fluid. It was not conducive to the mechanism studies of HPG destabilization, etc. In this case, the as-prepared simulated fracturing fluid-0 was taken as the research object during the following mechanism study and experimental process in order to exclude some influencing factors. It is noteworthy that only when the cross-linked HPG were decomposed into small molecules can it be beneficial to the treatment of fracturing fluid and reduce formation damage in practical engineering applications. As illustrated in Fig 3, XPS measurement was used to further investigate the chemical components of the simulated fracturing fluid-0 and simulated fracturing fluid. Fig 3A and 3D display the XPS survey spectrum of the simulated fracturing fluid-0 and simulated fracturing fluid, respectively. It can be seen that the elements were detected such as C, O, N, etc. Fig 3B and 3E correspond to the bonding of C 1s of simulated fracturing fluid-0 and simulated fracturing fluid, respectively. The C 1s spectrum could be decomposed into three peaks at~284.8, 286.3 and 287.5 eV, corresponding to C–C, C–O and C = O groups, respectively [37]. After adding the breaker, the peak area ratio of C–C increased from 38% to 45%, C = O increased from 10% to 18% and C–O decreased from 52% to 37%. In addition, from the high-solution XPS spectra for N 1s (Fig 3F), whereas a new functional group of C–N at~397.8 eV appeared after the addition of breaker, as compared to that of simulated fracturing fluid-0 (Fig 3C) [38]. This indicated that the hydroxyl radicals and carbon-oxygen bonds were oxidized in the HPG molecules, leading to cleavage of the HPG molecular chain, which reduced the viscosity of the simulated fracturing fluid.

**Fig 4:**
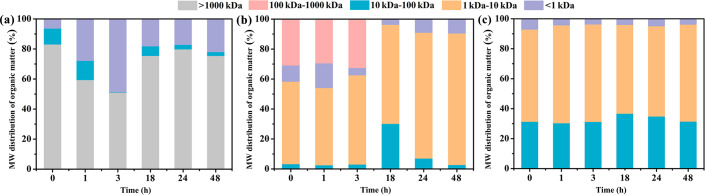
Molecular weight (MW) distributions of organic matter in the (a) pure HPG (b) simulated fracturing fluid-0 and (c) simulated fracturing fluid. Five MW fractions (<1 kDa, 1–10 kDa, 10–100 kDa, 100–1000 kDa, and>1000 kDa) were identified in the pure HPG, simulated fracturing fluid-0 and simulated fracturing fluid.

Guar gum is a natural high molecular polymer, which is likely to be decomposed under certain light conditions or the presence of certain microorganisms. Therefore, it is necessary to investigate the decomposition of guar gum under natural conditions. Fig 4 exhibits the changes in the MW distributions of soluble organic matter in the pure HPG, simulated fracturing fluid-0 and simulated fracturing fluid, respectively. For a pure HPG solution, the proportion of maximum MW (>1000 kDa) in the soluble organic matter significantly decreased in the first three hours, which indicated that part of the HPG should be decomposed into small molecules (Fig 4A). A strange phenomenon apparent from Fig 4A was that the proportion of maximum MW increased starting from the eighteenth hour, which inferred that samples were placed overnight resulting in molecular aggregation. As shown in Fig 4B and 4C, the low MW (1–10 kDa) of organic substance represented the largest proportion for the simulated fracturing fluid-0 and simulated fracturing fluid within 48 h. Notably, there were no maximum MW (>1000 kDa) of organic substance because of adding breaker in the simulated fracturing fluid. In general, degradation of soluble organic substance was distinct in 18–48 h in the simulated fracturing fluid-0. Furthermore, the change of MW distribution was not obvious as a function of time in the simulated fracturing fluid, and MW of approximately 70% organic substance was less than 10 kDa.

### 3.3. E-DLVO model

The wonderful process of simulated fracturing fluid from turbid to clear encouraged us to further study the destabilization performance of HPG. As is well known, the DLVO theory was developed by Derjaguin, Landau, Verwey and Overbeek [39, 40]. The theory reckons that the interaction of attractive and repulsive forces exits near the surface of a charged colloidal in an electrolyte solution [41–43]. This theory is relatively perfect in estimating trends in the mobility of numerous colloidal particles of the environment, especially in the stability of colloidal particles and the flocculating mechanism of flocculant to colloidal particles [44, 45]. Given that, we could further study about how HPG maintains a steady state in high-salt environment of fracturing fluid and seek the method of effective destabilization through the discussion of the DLVO model. This theory holds that colloidal particles are often mutually attractive and exclusive due to van der Waals interaction energy and the intersection of electrical double layer between colloidal particles, respectively [46, 47]. The van der Waals force (*V*_*A*_) among each particle was defined by the equation [48]:

$$V_{A}=-A_{131}R/12H$$ (1)

where we had the effective Hamaker constant (*A*_131_) given by the equation below:

A131≈(A1112−A3312)2 (2)

where *H* is the operating distance of interfacial forces, *R* is the median particle size, and *A*_131_ represents effective Hamaker constant of particle in medium. *A*_11_ and *A*_33_ are the Hamaker constant of particle and medium in a vacuum, respectively. Besides, the electrostatic repulsion energy (*V*_*R*_) was calculated according to the following equation:

$$V_{R}=2\Phi \varepsilon R\phi_{0}^{2}\;\text{ln}\;\left[1+\exp\left(-kH\right)\right]$$ (3)

where *ε* is the permittivity of solution, $\Phi_{0}$ is the particle surface potential, *R* is half of the median particle size, and *k* is Debye constant whose reciprocal is the thickness of double layer. Moreover, Debye constant (*k*) could be defined by the equation below:

$$k=\frac{4e^{2}N_{A}I}{\varepsilon k_{B}T}$$ (4)

where *N*_*A*_ is the Avogadro constant, *I* is the ionic strength, and *k*_*B*_ is the Boltzmann constant. However, the interparticle interactions were far more complicated than ordinary colloidal dispersion systems in the actual flocculation process. Classical DLVO theory also cannot explain the aggregation and dispersion behavior of colloidal particles in these systems. Therefore, extended DLVO (E-DLVO) theory was proposed on the basis of DLVO theory. On the DLVO potential curve of particle interaction, the total energy of particles interaction (*V*_*T*_) can be explained as follows:

$$V_{T}=V_{A}+V_{R}$$ (5)

HPG structure was similar to cellulose, and contained a large amount of -OH, -COOH and other hydrophilic groups. These hydrophilic groups absorbed several times or even dozens of times water of their own weight through hydration in an aqueous solution form a relatively stable hydration layer structure and bind water molecules within this range, which have good water retention property. Evidently, the most effective point that break its stability was damaging its hydration. The hydrophobic attraction energy between the hydrophobic colloidal particles could be attributed to the interface polar interaction particle between colloidal particles [49]. For the surface of hydrophilic particles or the surface of hydrophilic particles adsorbing chemicals, the polarizing effect of surface polar regions to adjacent water molecules formed a hydration force. The parameters of HPG E-DLVO model were summarized in Table 3.

**Table 3:** The parameter values of HPG E-DLVO model.

Parameters	Values
$\Phi_{0}$ (mV)	7.00 × 10^−3^
*ε* (C·V^−1^·m^−1^)	7.08 × 10^−10^
*A*_11_ (J)	6.30 × 10^−20^
*A*_33_ (J)	4.84 × 10^−20^
*R* (m)	6.50 × 10^−7^
*k*_*B*_ (J·K^−1^)	1.38 × 10^−23^
*I* (mol·L^−1^)	0.23
*V*_*A*_	−5.03/H
*V*_*R*_	14.16 ln(1 + e^−0.0462H^)

**Fig 5:**
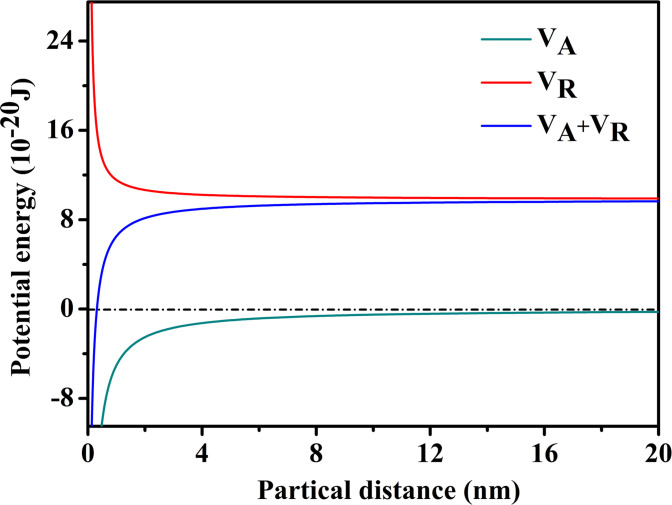
Interaction energy curves between HPG colloidal particles.

Interaction energy curves between HPG colloidal particles are presented in Fig 5. *V*_*A*_ and *V*_*R*_ were all zero when the interparticle distance was far away. *V*_*R*_ worked primarily when the interparticle distance was 20 nm. Furthermore, *V*_*A*_ did not work until the particles overcame *V*_*R*_, and continued to approach a certain distance, at which time *V*_*R*_ was much larger than *V*_*A*_. Therefore, the way to destabilize HPG colloid was that reducing the electrostatic repulsion energy between the colloidal particles, in which the colloidal particles were aggregated and agglomerated by van der Waals force, and separated from the fracturing fluid.

### 3.4. Analysis of coagulation and precipitation process

**Fig 6:**
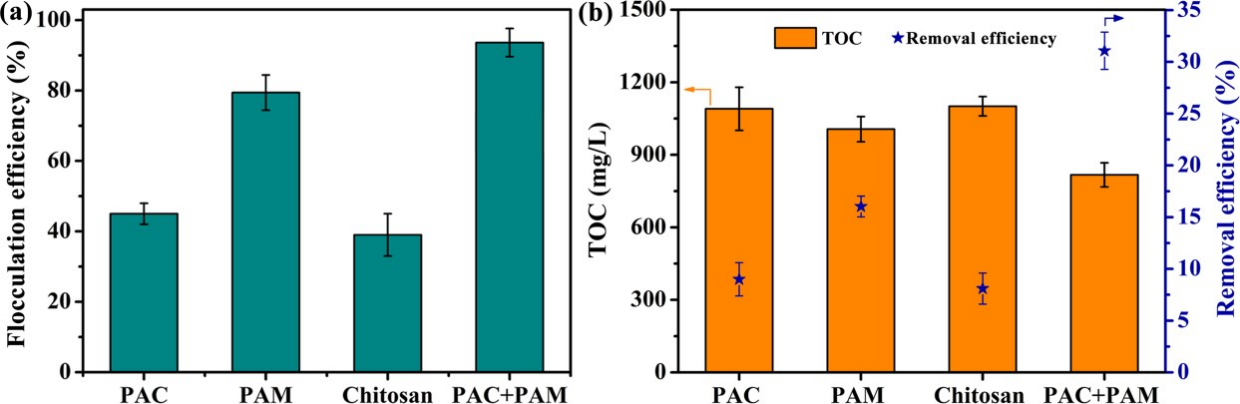
(a) The flocculation efficiency, (b) TOC content and removal efficiency of different flocculants after treating simulated fracturing fluid.

The flocculation treatment is the most common treatment technology in the area of oilfield wastewater treatment, because of its easy operation and low cost. To be specific, the flocculants realized the flocculation and sedimentation of the fracturing fluid containing massive colloidal particles and other impurities through the compression of the electric double layer, charge neutralization, adsorption bridging mechanisms and trap precipitation. More explicitly, coagulation was achieved through the flocculation of clever combination of different types of flocculants. It is widely believed that adsorption bridging and charge neutralization play a major role for organic flocculant, and these mechanisms including compression of electric double layers, adsorption bridging and trap precipitation play an important role for inorganic or natural polymer flocculant. During the flocculation process, the colloidal particles suspended in the system were destabilized, collided and condensed into larger flocs which then separated from the system by precipitation due to the addition of the flocculant. The flocculation efficiency and TOC removal efficiency of different flocculants for treating the simulated fracturing fluid are displayed Fig 6A and 6B, respectively. In contrast to other types of flocculants, PAM had high flocculation effect for the simulated fracturing fluid, which formed larger flocs and precipitated easily (Fig 6A). In order to further determine the effect of flocculation on the removal of HPG, TOC content and removal efficiency of HPG after flocculation are shown in Fig 6B. These results further confirmed that PAM possessed satisfactory flocculation effect, and the combination of PAC and PAM facilitated to achieve the best flocculation effect. From the above, it is reasonable to conclude that this flocculation method combining inorganic and organic flocculant should be selected to achieve a better flocculation effect in the flocculation process.

## 4. Conclusion

In summary, we designed the facile, efficient and economical HPG removal technology through a series of processes including gel-breaking, coagulation and precipitation towards the treatment of fracturing fluids. Combination of different types of flocculants achieved the HPG removal by compressing the electric double layer, charge neutralization, adsorption bridging mechanisms and trap precipitation, which make colloidal particles form floc to precipitate and separate from the fracturing fluids. The above approach is facile and low-cost easy to promote, avoiding the complicated treating process and use of complex equipment. Simultaneously, our research extends the knowledge into the explanations of aggregation and dispersion behavior between colloidal particles in fracturing fluid on the basis of DLVO theory. More importantly, this essay has argued that is the best instrument to a combination of PAC and PAM during the coagulation process. From the above, these studies thus offer a new strategy to treat the removal of HPG from fracturing fluid under the high-salt complex water environment.

## Supporting information

S1 Fig The digital photos of simulated fracturing fluid (a) before and (b) after heating.
(DOCX)

S2 Fig EDX Mapping results of the other elements of original fracturing fluid: Zr, Pu, Nb and Si. (DOCX)

S1 Graphical abstract (DOCX)

## References

[1] Jafry HR, Pasquali M, Barron AR. Effect of functionalized nanomaterials on the rheology of borate cross-linked guar gum. Ind Eng Chem Res. 2011;50(6):3259–3264. 10.1021/ie101836z

[2] Zhang W, Liu N, Cao Y, Chen Y, Xu L, Lin X, et al. A solvothermal route decorated on different substrates: controllable separation of an oil/water mixture to a stabilized nanoscale emulsion. Adv Mater. 2015;27(45):7349–7355. 10.1002/adma.201502695 26489016

[3] Gao S, Sun J, Liu P, Zhang F, Zhang W, Yuan S, et al. A robust polyionized hydrogel with an unprecedented underwater anti-crude-oil-adhesion property. Adv Mater. 2016;28(26):5307–5314. 10.1002/adma.201600417 27159880

[4] Patra AS, Ghorai S, Ghosh S, Mandal B, Pal S. Selective removal of toxic anionic dyes using a novel nanocomposite derived from cationically modified guar gum and silica nanoparticles. J Hazard Mater. 2016;301:127–136. 10.1016/j.jhazmat.2015.08.042 26348145

[5] Gao SJ, Zhu YZ, Zhang F, Jin J. Superwetting polymer-decorated SWCNT composite ultrathin films for ultrafast separation of oil-in-water nanoemulsions. J Mater Chem A. 2015;3(6):2895–2902. 10.1039/c4ta05624h

[6] Munirasu S, Haija MA, Banat F. Use of membrane technology for oil field and refinery produced water treatment—A review. Process Saf Environ. 2016;100:183–202. 10.1016/j.psep.2016.01.010

[7] Quosay AA, Knez D, Ziaja J. Hydraulic fracturing: New uncertainty based modeling approach for process design using Monte Carlo simulation technique. PLOS ONE. 2020;15(7):e0236726. 10.1371/journal.pone.0236726 32726370 PMC7390409

[8] Zhang F, Ma G, Liu X, Tao Y, Feng D, Li R. Experimental analysis of multiple factors on hydraulic fracturing in coalbed methane reservoirs. PLOS ONE. 2018;13(4):e0195363. 10.1371/journal.pone.0195363 29621295 PMC5886525

[9] Hurnaus T, Plank J. Behavior of titania nanoparticles in cross-linking hydroxypropyl guar used in hydraulic fracturing fluids for oil recovery. Energ Fuel. 2015;29(6):3601–3608. 10.1021/acs.energyfuels.5b00430

[10] Pu W-f, Du D-j, Liu R. Preparation and evaluation of supramolecular fracturing fluid of hydrophobically associative polymer and viscoelastic surfactant. J Petrol Sci Eng. 2018;167:568–576. 10.1016/j.petrol.2018.04.032

[11] Yan Z, Dai C, Zhao M, Sun Y, Zhao G. Development, formation mechanism and performance evaluation of a reusable viscoelastic surfactant fracturing fluid. J Ind Eng Chem. 2016;37:115–122. 10.1016/j.jiec.2016.03.012

[12] Kekacs D, Drollette BD, Brooker M, Plata DL, Mouser PJ. Aerobic biodegradation of organic compounds in hydraulic fracturing fluids. Biodegradation. 2015;26(4):271–287. 10.1007/s10532-015-9733-6 26037076

[13] Vidic RD, Brantley SL, Vandenbossche JM, Yoxtheimer D, Abad JD. Impact of shale gas development on regional water quality. Science. 2013;340(6134):1235009. 10.1126/science.1235009 23687049

[14] Murali Mohan A, Hartsock A, Bibby KJ, Hammack RW, Vidic RD, Gregory KB. Microbial community changes in hydraulic fracturing fluids and produced water from shale gas extraction. Environ Sci Technol. 2013;47(22):13141–13150. 10.1021/es402928b 24088205

[15] Rogers JD, Burke TL, Osborn SG, Ryan JN. A framework for identifying organic compounds of concern in hydraulic fracturing fluids based on their mobility and persistence in groundwater. Environmental Science & Technology Letters. 2015;2(6):158–164. 10.1021/acs.estlett.5b00090

[16] Adgate JL, Goldstein BD, McKenzie LM. Potential public health hazards, exposures and health effects from unconventional natural gas development. Environ Sci Technol. 2014;48(15):8307–8320. 10.1021/es404621d 24564405

[17] Bahamdan A, Daly WH. Hydrophobic guar gum derivatives prepared by controlled grafting processes–Part II: rheological and degradation properties toward fracturing fluids applications. Polym Advan Technol. 2007;18(8):660–672. 10.1002/pat.875

[18] Sari MA, Chellam S. Mechanisms of boron removal from hydraulic fracturing wastewater by aluminum electrocoagulation. J Colloid Interface Sci. 2015;458:103–111. 10.1016/j.jcis.2015.07.035 26210100

[19] Dai C, Wang K, Liu Y, Fang J, Zhao M. Study on the reutilization of clear fracturing flowback fluids in surfactant flooding with additives for Enhanced Oil Recovery (EOR). PLOS ONE. 2014;9(11):e113723. 10.1371/journal.pone.0113723 25409507 PMC4237460

[20] Thomas L, Tang H, Kalyon DM, Aktas S, Arthur JD, Blotevogel J, et al. Toward better hydraulic fracturing fluids and their application in energy production: A review of sustainable technologies and reduction of potential environmental impacts. J Petrol Sci Eng. 2019;173:793–803. 10.1016/j.petrol.2018.09.056

[21] Alzobaidi S, Lee J, Jiries S, Da C, Harris J, Keene K, et al. Carbon dioxide-in-oil emulsions stabilized with silicone-alkyl surfactants for waterless hydraulic fracturing. J Colloid Interface Sci. 2018;526:253–267. 10.1016/j.jcis.2018.04.056 29747039

[22] Lester Y, Yacob T, Morrissey I, Linden KG. Can we treat hydraulic fracturing flowback with a conventional biological process? The case of guar gum. Environmental Science & Technology Letters. 2013;1(1):133–136. 10.1021/ez4000115

[23] Lester Y, Ferrer I, Thurman EM, Sitterley KA, Korak JA, Aiken G, et al. Characterization of hydraulic fracturing flowback water in Colorado: implications for water treatment. Sci Total Environ. 2015;512–513:637–644. 10.1016/j.scitotenv.2015.01.043 25658325

[24] Hickenbottom KL, Hancock NT, Hutchings NR, Appleton EW, Beaudry EG, Xu P, et al. Forward osmosis treatment of drilling mud and fracturing wastewater from oil and gas operations. Desalination. 2013;312:60–66. 10.1016/j.desal.2012.05.037

[25] Dai L, Long Z, Chen J, An X, Cheng D, Khan A, et al. Robust guar gum/cellulose nanofibrils multilayer films with good barrier properties. ACS Appl Mater Interfaces. 2017;9(6):5477–5485. 10.1021/acsami.6b14471 28102068

[26] Pan X, Wang Q, Ning D, Dai L, Liu K, Ni Y, et al. Ultraflexible self-healing guar gum-glycerol hydrogel with injectable, antifreeze, and strain-sensitive properties. ACS Biomaterials Science & Engineering. 2018;4(9):3397–3404. 10.1021/acsbiomaterials.8b00657 33435073

[27] Shang X, Ding Y, Wang Y, Yang L. Rheological and performance research on a regenerable polyvinyl alcohol fracturing fluid. PLOS ONE. 2015;10(12):e0144449. 10.1371/journal.pone.0144449 26641857 PMC4671606

[28] Gong H, Liu M, Chen J, Han F, Gao C, Zhang B. Synthesis and characterization of carboxymethyl guar gum and rheological properties of its solutions. Carbohyd Polym. 2012;88(3):1015–1022. 10.1016/j.carbpol.2012.01.057

[29] Hasan AMA, Abdel-Raouf ME. Applications of guar gum and its derivatives in petroleum industry: A review. Egyptian Journal of Petroleum. 2018;27(4):1043–1050. 10.1016/j.ejpe.2018.03.005

[30] Gastone F, Tosco T, Sethi R. Green stabilization of microscale iron particles using guar gum: bulk rheology, sedimentation rate and enzymatic degradation. J Colloid Interface Sci. 2014;421:33–43. 10.1016/j.jcis.2014.01.021 24594029

[31] Szopinski D, Kulicke WM, Luinstra GA. Structure-property relationships of carboxymethyl hydroxypropyl guar gum in water and a hyperentanglement parameter. Carbohydr Polym. 2015;119:159–166. 10.1016/j.carbpol.2014.11.050 25563956

[32] Lu X, Li Y, Feng W, Guan S, Guo P. Self-healing hydroxypropyl guar gum/poly (acrylamide-co-3-acrylamidophenyl boronic acid) composite hydrogels with yield phenomenon based on dynamic PBA ester bonds and H-bond. Colloids and Surfaces A: Physicochemical and Engineering Aspects. 2019;561:325–331. 10.1016/j.colsurfa.2018.10.071

[33] Thombare N, Mishra S, Siddiqui MZ, Jha U, Singh D, Mahajan GR. Design and development of guar gum based novel, superabsorbent and moisture retaining hydrogels for agricultural applications. Carbohydr Polym. 2018;185:169–178. 10.1016/j.carbpol.2018.01.018 29421054

[34] Kaith BS, Sharma R, Kalia S, Bhatti MS. Response surface methodology and optimized synthesis of guar gum-based hydrogels with enhanced swelling capacity. RSC Adv. 2014;4(76):40339–40344. 10.1039/c4ra05300a

[35] Dai L, Zhang L, Wang B, Yang B, Khan I, Khan A, et al. Multifunctional self-assembling hydrogel from guar gum. Chem Eng J. 2017;330:1044–1051. 10.1016/j.cej.2017.08.041

[36] Balachandramohan J, Sivasankar T, Sivakumar M. Facile sonochemical synthesis of Ag_2_O-guar gum nanocomposite as a visible light photocatalyst for the organic transformation reactions. J Hazard Mater. 2019;385:121621. 10.1016/j.jhazmat.2019.121621 31784127

[37] Sun Y, Wu ZY, Wang X, Ding C, Cheng W, Yu SH, et al. Macroscopic and Microscopic Investigation of U(VI) and Eu(III) Adsorption on Carbonaceous Nanofibers. Environ Sci Technol. 2016;50(8):4459–4467. 10.1021/acs.est.6b00058 26998856

[38] Liu S, Chen F, Li S, Peng X, Xiong Y. Enhanced photocatalytic conversion of greenhouse gas CO_2_ into solar fuels over g-C_3_N_4_ nanotubes with decorated transparent ZIF-8 nanoclusters. Applied Catalysis B: Environmental. 2017;211:1–10. 10.1016/j.apcatb.2017.04.009

[39] Verwey EJW, Overbeek JTG. Theory of the stability of lyophobic colloids. Journal of Colloid Science. 1955;10(2):224–225. 10.1016/0095-8522(55)90030-1

[40] Derjaguin B, Landau L. Theory of the stability of strongly charged lyophobic sols and of the adhesion of strongly charged particles in solutions of electrolytes. Prog Surf Sci. 1993;43(1–4):30–59. 10.1016/0079-6816(93)90013-l

[41] Yuan R, Zhang W, Tao X, Wang S, Zhang L. Coupled effects of high pH and chemical heterogeneity on colloid retention and release in saturated porous media. Colloids and Surfaces A: Physicochemical and Engineering Aspects. 2020;586:124285. 10.1016/j.colsurfa.2019.124285

[42] Sanaei A, Tavassoli S, Sepehrnoori K. Investigation of modified water chemistry for improved oil recovery: Application of DLVO theory and surface complexation model. Colloids and Surfaces A: Physicochemical and Engineering Aspects. 2019;574:131–145. 10.1016/j.colsurfa.2019.04.075

[43] Adamczyk Z, Weroński P. Application of the DLVO theory for particle deposition problems. Adv Colloid Interfac. 1999;83(1–3):137–226. 10.1016/s0001-8686(99)00009-3

[44] Kuhnen F, Barmettler K, Bhattacharjee S, Elimelech M, Kretzschmar R. Transport of iron oxide colloids in packed quartz sand media: monolayer and multilayer Deposition. J Colloid Interface Sci. 2000;231(1):32–41. 10.1006/jcis.2000.7097 11082245

[45] Tosco T, Bosch J, Meckenstock RU, Sethi R. Transport of ferrihydrite nanoparticles in saturated porous media: role of ionic strength and flow rate. Environ Sci Technol. 2012;46(7):4008–4015. 10.1021/es202643c 22356610

[46] Nikoo AH, Malayeri MR. Incorporation of surface energy properties into general crystallization fouling model for heat transfer surfaces. Chem Eng Sci. 2020;215:115461. 10.1016/j.ces.2019.115461

[47] Cao T, Trefalt G, Borkovec M. Aggregation of colloidal particles in the presence of hydrophobic anions: importance of attractive non-DLVO forces. Langmuir. 2018;34(47):14368–14377. 10.1021/acs.langmuir.8b03191 30383385

[48] Shen C, Bradford S, Wang Z, Huang Y, Zhang Y, Li B. DLVO interaction energies between hollow spherical particles and collector surfaces. Langmuir. 2017;33(40):10455–10467. 10.1021/acs.langmuir.7b02383 28925268

[49] Coyne J, Zhao N, Olubode A, Menon M, Wang Y. Development of hydrogel-like biomaterials via nanoparticle assembly and solid-hydrogel transformation. J Control Release. 2020;318:185–196. 10.1016/j.jconrel.2019.12.026 31857102 PMC6954609

