# Supplementary material for: Design of facile technology for the efficient removal of hydroxypropyl guar gum from fracturing fluid
Source: PLoS One. 2021 Mar 4;16(3):e0247948. doi: 10.1371/journal.pone.0247948 (PMC7932517; doi:10.1371/journal.pone.0247948)
Supplement: S1 Fig — The digital photos of simulated fracturing fluid (a) before and (b) after heating. (DOCX) [file pone.0247948.s001.docx]

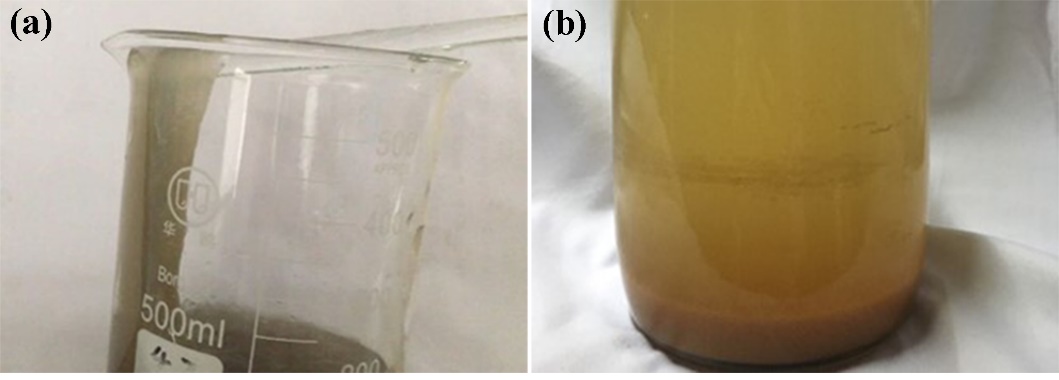


S1 Fig. The digital photos of simulated fracturing fluid (a) before and (b) after heating.

The simulated fracturing fluid had a very high viscosity before heating, which can be stirred up with a glass rod. After heating, the color of the simulated fracturing fluid was yellow, with a layer of dark sediment at the bottom, which had a lower viscosity.
