# Supplementary figures and images for: Design of facile technology for the efficient removal of hydroxypropyl guar gum from fracturing fluid
Source: PLoS One. 2021 Mar 4;16(3):e0247948. doi: 10.1371/journal.pone.0247948 (PMC7932517; doi:10.1371/journal.pone.0247948)

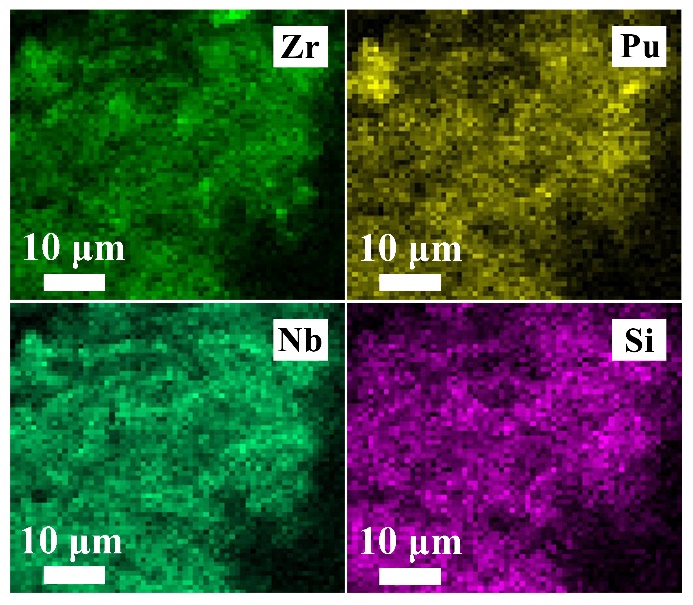


S2 Fig. EDX Mapping results of the other elements of original fracturing fluid: Zr, Pu, Nb and Si.

Supplement: S2 Fig — (DOCX) [file pone.0247948.s002.docx]
