# Supplementary material for: Design of facile technology for the efficient removal of hydroxypropyl guar gum from fracturing fluid
Source: PLoS One. 2021 Mar 4;16(3):e0247948. doi: 10.1371/journal.pone.0247948 (PMC7932517; doi:10.1371/journal.pone.0247948)
Supplement: S1 Graphical abstract — (DOCX) [file pone.0247948.s003.docx]

**Graphical Abstract**


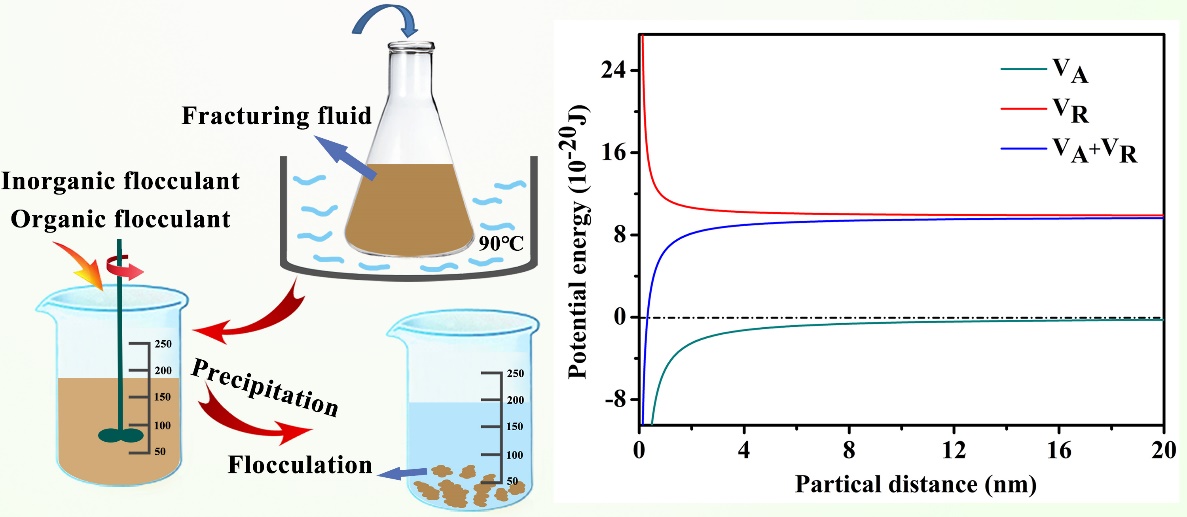


We designed a facile and economical technology for the efficient removal of HPG from fracturing fluid, which also explored and studied the destabilization mechanism of HPG on the basis of DLVO theory.
